# Research Progress of DNA Methylation Markers for Endometrial Carcinoma Diagnosis

**DOI:** 10.7150/jca.104214

**Published:** 2025-01-01

**Authors:** Haoning Zheng, Cuisong Yu, Lu Yang, Fenghua Zhou, Aijun Liu

**Affiliations:** 1 Clinical Pathology Department, Shandong Second Medical University, Shandong Province, Weifang, Shandong 261042, P.R. China.; 2 Department of Pathology, The seventh Medical Center, Chinese PLA General Hospital, Beijing, 100700, P.R. China.; 3 Department of Gynecology and Obstetrics of Qingdao West Coast New Area People's Hospital, Shandong Province, Qingdao, Shandong 266000, P.R. China.

**Keywords:** endometrial carcinoma, DNA methylation, epigenetics, diagnostic biomarkers

## Abstract

Endometrial carcinoma (EC) is the most common malignancies of the female reproductive system in developed countries and areas. Ultrasound-guided and hysteroscopic samplings are commonly used to diagnose EC. However, clinicians question their diagnostic efficacy and the associated patient discomfort. DNA methylation is the widely studied epigenetic alteration in human tumors, and tumor screening and diagnosis. This review summarized common methods for collecting clinical samples for methylation testing. Furthermore, we analyzed the diagnostic evaluation indices of different methylation marker assays in clinical diagnosis and discussed the challenges of methylation testing in the future application of EC diagnosis.

## 1. Introduction

Endometrial carcinoma (EC) is the most common malignancy in the female reproductive system in developed countries. According to Global Cancer Statistics 2020, EC is the sixth most common cancer in women, with a global incidence of 417,000 new cases, mostly prevalent between the ages of 65 and 75^1^, ^2^. Over the past three decades, EC diagnoses have increased by 132% globally, and deaths have nearly doubled^3^.

In 1983, Oncol proposed the traditional classification of EC; estrogen-dependent EC (Type I EC) is associated with obesity, hyperlipidemia, and excessive estrogen Estrogen-independent EC (Type II EC) occurs without the aforementioned etiological factors^4^. Type I EC is represented by endometrioid carcinoma, which is characterized by a high degree of differentiation, generally favorable prognosis, slow tumor growth, and low grade and invasiveness. Type II EC encompasses various histological types, including serous, clear cell, and mixed carcinomas, and other rare types. Type II ECs typically exhibit poor prognosis, rapid tumor growth, and high invasiveness, often presenting with metastasis at the time of diagnosis^5^. In 2013, The Cancer Genome Atlas project conducted a large-scale molecular characterization of and proposed a new classification method based on tumor genetic mutations, copy number variations, mRNA expression, methylation profiles, and protein expression. There are four molecular subtypes. The POLE ultramutated type is characterized by a high mutation rate due to mutations in the POLE gene. Microsatellite instability (MSI) hypermutated type exhibits a high level of MSI and a moderate mutation rate. The copy number low (endometrioid) type displays low copy number alterations and is often associated with Type I EC^6^. The copy number high (serous-like) type is characterized by high copy number alterations and is typically correlated with Type II EC and a poorer prognosis. The molecular classification of EC allows clinicians to select appropriate treatments improving patient prognosis^5^, ^7^.

Early diagnosis of EC is associated with better prognostic outcomes; therefore, accurate diagnosis and timely treatment are crucial for its management^8^. Transvaginal ultrasound (TVU) is the preferred early screening and diagnosis option. A study of over 1,000 patients showed that an endometrial thickness of ≥ 5 mm in postmenopausal women had a sensitivity of 96.2% and a negative predictive value of 99.3% for detecting EC. However, TVU specificity was only 51.5%, indicating the need for additional screening to rule out other malignancies^9^. Hysteroscopy allows the direct sampling of suspicious lesions and can be used in TVU-positive and recurrently symptomatic women. Information from endometrial biopsy is often used for preoperative disease staging, is essential for surgical management, and guides the scope of the procedure. However, hysteroscopy also increases the risk of cancer spread, and physical discomfort and false-negative results are common complications^10^, ^11^.

In recent years, AI has shown extensive potential for application in the diagnosis of EC, particularly in areas of pathologic analysis, molecular diagnosis, predictive model construction, and so on. For example, Li *et al.* developed an artificial intelligence system for screening and diagnosing EC, successfully classifying malignant and benign EC cells^12^. AI could also provide more precise assessments in the molecular classification of EC^13^. Consequently, safer, more effective, and reliable means of screening and early diagnosis in high-risk populations is needed to reduce lethality, improving the clinical prognosis of EC.

**Figure 1:**
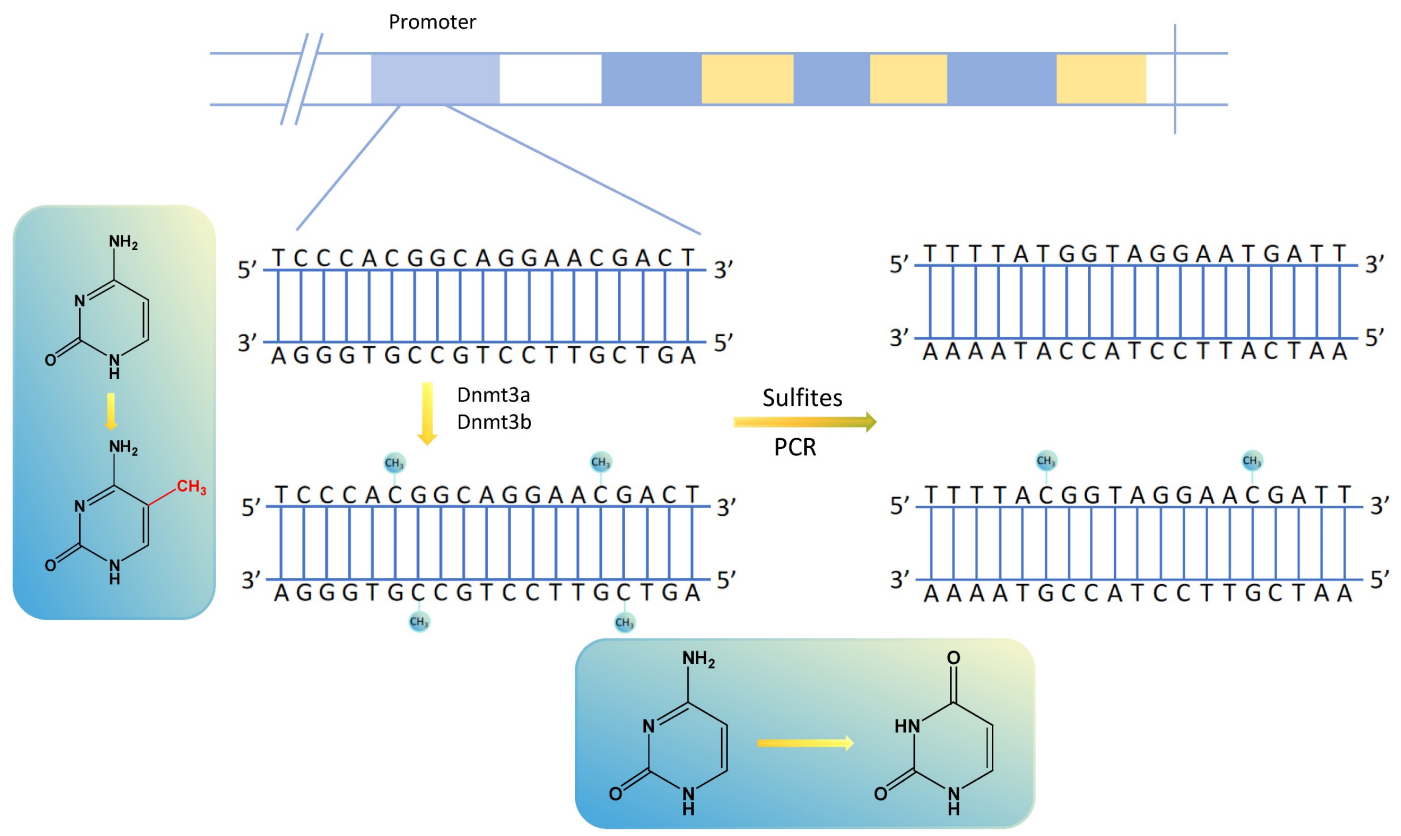
Altered gene methylation sites and gene changes after PCR amplification by sulfite treatment.

Methylation transfers methyl groups from active methyl compounds to other compounds and occurs in specific proteins or nucleic acids chemically modified to form methylated products^14^, ^15^. DNA methylation, in which methyltransferases catalyze the transfer of active methyl groups to target chemicals without altering DNA sequence composition, is an important type of epigenetic regulation^16^. It usually occurs in the promoter region of the DNA sequence, and its aberrant expression can result in the aberrant expression of tumor-associated genes in various human tumors^17^, ^18^. Among the DNA methylation sites, the most prominent manifestation is using DNA methyltransferases to transfer methyl groups to cytosine 5 carbon atom of cytosine-phosphate-guanine (CpG) dinucleotides to form 5-methylcytosine (5mC), interfering with promoter recognition and gene regulation^19^. The regions of aggregated CpG dinucleotides are called CpG islands, and the promoter regions of genes usually contain many CpG islands. Methylated cytosine is unrecognized by sulfite, determining whether and to what extent the DNA is methylated^18^ (Fig. 1). There are many ways to detect DNA methylation, including genome-wide methylation detection technologies (WGBS, RRBS, Illumina EPIC BeadChip Microarray, MeDIP-seq and MethylRAD) and site-specific methylation detection technologies (pyrophosphate sequencing, Massarray, BSP and MSP)^20^-^22^.

EC is diagnosed by detecting promoter methylation levels of single or multiple genes in the specimen of endometrium^23^. Genes with aberrant DNA methylation interfere with various biological pathways, such as cell adhesion and proliferation, cell cycle regulation, and apoptosis, contributing to the development and progression of EC^24^. Big data and bioinformatics have revealed that methylation markers can help predicting less aggressive tumors and are suitable for fertility preservation therapy^25^. This review summarized the commonly used clinical tests for methylation sample collection, genes or genomes that play a key role in the organism, and the diagnostic evaluation of methods for detecting methylation in clinical EC diagnostic applications. We also explored the prospects for applying DNA methylation in EC screening and diagnosis.

## 2. DNA methylation markers for EC

The development and progression of EC involve multiple biological processes and numerous genes. Gene methylation plays a crucial role in regulating the expression of related genes or controlling other genes. By focusing on the CpG islands within gene promoter regions and utilizing next-generation sequencing technology supported by a large number of clinical samples, the methylation status of some specific genes can be observed. From the perspective of EC etiology, we collected data on methylation-related genes in EC and clinical diagnostic information for certain genes to explore their feasibility as early diagnostic markers for EC (Table 1).

### 2.1 Genes involved in regulating cell proliferation and differentiation

Ras-binding domain family 1 isoform A (RASSF1A) regulates cellular processes such as cell cycle arrest, migration, microtubule stabilization, and pro-apoptosis in response to various stimuli^26^. Hypermethylation of the RASSF1A promoter is frequently associated with poor prognostic characters, including advanced clinical stage, lymph node and/or distant metastasis, and drug resistance^27^. RASSF1A may play a role in cellular proliferation and apoptosis via regulating the RAS-MAPK signaling pathway^28^. Pijnenborg *et al.* reported that 85% of patients with EC exhibited RASSF1A methylation, and RASSF1A promoter methylation was present in 70% of cases in premenopausal EC^29^. Fiolka *et al.* found a significant correlation between RASSF1A and higher tumor grade, deeper infiltration of the uterine myometrium, and metastases in the pelvic lymph nodes^30^. As a therapeutic target, 5-aza-2'-deoxycytidine (5-Aza-CdR) may reverse the methylation status of the RASSF1A gene, restored its mRNA and protein expression, and control the growth of EC cell lines by inducing apoptosis^31^.

The calcineurin 13 (CDH13) gene is a novel member of the calcineurin superfamily. It is primarily expressed on transmembrane glycoproteins on the surface of epithelial cells and mediates intercellular Ca2^+^dependent adhesion to maintain normal tissue structure^32^. CDH13 expression in many tumor cell lines inhibits cell proliferation and invasiveness, increases susceptibility to apoptosis, and reduces tumor growth *in vivo* models^33^. CDH13 hypermethylation is an independent prognostic factor in EC. Early reports have identified significant changes in the methylation of the CDH13 promoter during the development and progression of EC^34^. Yan Sheng and colleagues demonstrated that treatment with 5-Aza-CdR or trichostatin A partially reversed mRNA levels, proving that the methylation of CDH13 is one of the main reasons for its decreased expression in cancer cells^35^. Clinical analysis of EC samples revealed that the methylation level of the CDH13 promoter ranged from 80% to 81.36%, while in atypical hyperplasia samples, it ranged from 50% to 51.7%^36^, ^37^. Krasnyi's team found that cysteine dioxygenase type 1 (CDO1) and CDH 13 gene methylation levels in EC tissue samples (stage IA) predicted the outcome of drug treatment^38^.

Heart and neural crest derivatives expressed 2 (HAND2) gene encode a transcription factor that belongs to the basic Helix-Loop-Helix (bHLH) family. It is primarily expressed in cardiac development and neural crest derivatives^39^. HAND2 is crucial in regulating embryonic development, cell proliferation, and differentiation, particularly in the development of the right ventricle of the heart, neural crest cells, and limb buds^40^. HAND2 is present in endometrial stromal cells, where it inhibits ligand-dependent transcriptional activation of estrogen receptor alpha (ERα) and activates interleukin-15 transcription^41^. Reportedly, increased methylation of HAND2 is a hallmark of endometrial precancer, typically correlated with decreased RNA and protein levels. Women with high levels of HAND2 methylation respond less effectively to progesterone therapy for endometrial precancer^42^.

The bHLH Family Member e22 (BHLHE22) is a transcriptional repressor and regulates cell differentiation during neuronal development^43^. The expression of BHLHE22 protein was significantly lower in EC than in normal endometrium. High expression of BHLHE22 correlated with the MSI subtype, tumor grade, and patient age, and significantly improved survival outcomes^44^, ^45^. Furthermore, BHLHE22 overexpression inhibited the proliferation and migration of EC cells^45^. Phui-Ly Liew discovered that the highly methylated panels BHLHE22/CDO1/HAND2 (87.0% sensitivity and 86.0% specificity) and BHLHE22/CDO1/TBX5 (89.1% sensitivity and 80.0% specificity) exhibited significant differences, effectively distinguishing between benign and malignant endometrial lesions^46^. Rui-Lan Huang used methylation-specific PCR (QMSP) on 146 cervical scrapings and found that a panel consisting of any two of the three highly methylated genes BHLHE22, CDO1, and CELF4 demonstrated a sensitivity of 91.8% and a specificity of 95.5%^31^. In a recent study, the team led by Kuo-Chang Wen utilized MPap detection technology and observed that the sensitivity and specificity of the genes CDO1 / BHLHE22 in EC were above 90% and 70%, respectively^47^.

As an oncogene, the tumor suppressor activity of phosphatase and tensin homolog (PTEN) primarily depends on its lipid phosphatase function, inhibiting PI3K/AKT activation. Consequently, PTEN regulates various cellular processes, including proliferation, survival, energy metabolism, cell structure, and motility^48^. Khatami's team observed PTEN promoter methylation in 52.0% of EC tumor tissues, compared to 13.6% in non-tumor tissues^49^. Gotoh *et al.* employed DNA methylome microarray sequencing and found PTEN mutations and clonal expansion of tumor cells in atypical hyperplasia samples^50^. Liew *et al.* also demonstrated that PTEN mutations were seldomly present in cervical scrapings of normal endometrium (25%) and benign uterine lesions (10%), but adding PTEN mutation testing to the BHLHE22/CDO1-based methylation assay did not enhance the detection efficiency of EC^46^.

Adenomatous polyposis coli (APC) is a gene that disrupts the Wnt/β-catenin signaling pathway, preventing it from taking part in organ development, cell proliferation, survival, differentiation, and migration^51^. Zysman's team studied 114 endometrial adenocarcinoma specimens, and illustrated that the frequency of APC hypermethylation was increased in MSI+ endometrial tumors^52^. Ignatov *et al.* reported that DNA methylation frequency of the APC gene increased from atypical hyperplasia (23.5%) to early-stage EC (77.4%) and then gradually decreased in advanced carcinoma (24.2%)^53^. However, in a recent study, Lou *et al.* observed hypomethylation in the promoter of APC and upregulation of gene expression in mutant EC samples^54^.

The P16 gene belongs to the inhibitor of cyclin-dependent kinase 4 (INK4) gene family. It consists of four members, p16 INK4A, p15 INK4B, p18 INK4C, and p19 INK4D, and possesses biological properties of cell growth inhibition and tumor suppression^55^. Moreover, p16 inhibits cell cycle protein-dependent kinases, leading to G1 cell cycle arrest, whereas methylated p16 leads to tumor development^56^, ^57^. In a meta-analysis including 264 cases of EC patients, hypermethylation of the p16 gene promoter was associated with an increased risk of EC^58^. Multi-institutional studies have demonstrated that p16 methylation is rare in precancerous lesions, but predominant in advanced EC, and therefore is not indicated for early screening. However, it can be used as a potential prognostic marker^59^.

### 2.2 Genes related to hormone and metabolism

The imbalance of estrogen and progesterone is a significant cause of endometrial carcinogenesis, particularly in Type I EC. Hyperlipidemia and slow fat metabolism are also high-risk factors for EC.

CDO1 is a critical enzyme in cysteine catabolism and vital in physiological processes, including lipid metabolism, organismal growth, and development^60^. CDO1 enhances the production of reactive oxygen species to induce apoptosis. It interacts with peroxisome proliferator-activated receptor γ, activating the key tumor suppressor transcription factor CCAAT/enhancer-binding protein (C/EBP) α, thereby inhibiting tumor progression^61^, ^62^. CDO1 is a potential tumor suppressor gene. In cancers such as renal cell, breast, and colorectal cancer, the promoter region of CDO1 frequently undergoes hypermethylation, leading to reduced or absent expression^63^, ^64^. This methylation-induced silencing may allow cancer cells to evade cell death by oxidative stress, promoting tumor survival and progression^65^. Clinical data suggest that CDO1 is significantly hypermethylated in Types I and II of EC, and it can serve as a diagnostic marker to distinguish between cancerous and normal tissues^66^, ^67^.

Estrogen receptor 1 (ESR1) is the gene that encodes ERα. This nuclear receptor predominantly regulates gene expression by binding to estrogens, such as 17β-estradiol. It plays a vital role in the development of the reproductive system, bone health, cardiovascular function, and the growth and differentiation of breast tissue^68^, ^69^. SNAI2 promotes ESR1 methylation by recruiting DNA methyltransferase 3B rather than DNA methyltransferase 1 in ERα-positive breast cancer cells and may contribute to cell adhesion and junctions^70^. However, the role of ESR1 methylation in the development of EC remains controversial. Carla Bartosch and colleagues analyzed ERα and PRA promoter methylation in 45 cases of EC and concluded that the methylation of these genes plays a limited role in the etiology of the disease^71^. Vanessa Todorow demonstrated that in three out of five EC cell lines, the promoter region of ESR1 was methylated, suggesting that ESR1 methylation may influence EC development^72^. Results of bioinformatics analysis also support this viewpoint^73^, ^74^. PIWIL1 mediates the ERα signaling pathway involved in E2-stimulated carcinoma cell proliferation, which may be one of the mechanisms by which ESR1 methylation contributes to EC progression^75^.

### 2.3 Genes involved in expression regulation and epigenetics

The impairment of gene repair function and the body's immune system affect cancer progression in terms of prevention and elimination. Meanwhile, focusing on the methylation changes of the genes themselves, it is important to consider that alteration of genes that promote methylation can also impact EC development and progression. These genes have broader effects and can assist in the early diagnosis and prognostic assessment when used alongside methylation biomarkers.

The human mutL homolog 1 (hMLH1) gene undergoes DNA mismatch repair (MMR) gene mutations in Lynch syndrome, commonly used in the pathologic diagnosing of rectal carcinoma and ECs^6^, ^76^. Two-thirds of EC exhibit dMMR, mainly caused by methylation of the MLH1 promoter^77^. Three-quarters of EC patients aged 36-59 exhibited methylation of the MLH1 gene. Annukka Pasanen *et al.* found that 76% of 244 dMMR cases were associated with methylation^78^, ^79^. Kahn *et al.* analyzed that 86.3% (1016/1159) patients' loss of MLH1 staining were due to MLH1 methylation^80^. Clinical studies have found that tumor size is significantly associated with MLH1 methylation^81^, ^82^. Thus, hMLH1 methylation testing may be used as an early clinical screening and prognostic indicator for patients with EC^34^.

Methyl-CpG-binding protein 2 (MeCP2) is a protein essential to regulating gene expression and DNA methylation^83^. It was initially studied extensively for its involvement in Rett syndrome, a neurodevelopmental disorder that affects brain development^84^. MeCP2 binds to methylated CpG dinucleotides and interacts with other proteins to either repress or activate the expression of specific genes^85^. In EC research, MeCP2 is associated with decreased promoter methylation, leading to higher expression levels and promoting methylation of other genes. Gene mutations in MeCP2 are associated with a favorable prognosis^86^, ^87^. Yuning Xiong *et al.* discovered that MeCP2 specifically binds to and methylates the hMLH1 promoter^88^. Yongli Chu's team found that MeCP2 plays a key role in the silencing of the progesterone receptor-B (PR-B) gene, suggesting that epigenetic reactivation of PR-B could be explored as a potential strategy to sensitize PR-B-negative EC to progestin therapy^89^.

CUGBP Elav-like family member 4 (CELF4) is a member of the CELF protein family, which is involved in regulating processes such as alternative RNA splicing, post-transcriptional regulation, translation, and mRNA degradation^90^. Similar to CDO1 and BHLHE22, CELF4 was also first identified and reported by Huang *et al.* to exhibit abnormally elevated methylation in EC. Statistical analysis showed that CELF4 had a sensitivity of 96.0%, a specificity of 78.7%, and an AUC of 0.94^31^. In recent studies, researchers have shown a preference for the combined detection of CDO1 and CELF4 methylation, with sensitivity ranging from 84.9% to 87.5% and specificity from 86.6% to 95.9%^67^, ^91^, ^92^. Additionally, Zhao *et al.* and Kong *et al.* both suggested that combining BMI index and the joint use of TVU could improve the prediction of EC screening^67^, ^93^. However, a recent study also indicated that the combined use of CDO1 and CELF4 did not lead to better screening outcomes, with AUC values of only 0.6000 and 0.5286, respectively^94^.

## 3. Sample collection for DNA methylation detection

In populations with a low incidence of EC, TVU followed by endometrial biopsy is the most cost-effective strategy^95^. Surgical anesthesia can greatly reduce the pain associated with endometrial sampling. Nonetheless, it is difficult to ensure that patients can be painlessly sampled in outpatients for large-scale screening^96^. With the advancement in molecular methylation technology, the method of collecting vaginal fluid, urine, and blood samples to detect tumor methylation markers and understand their prognosis is gradually being applied in clinical diagnosis (Table 2).

### 3.1 Vaginal fluid collection

The collection of vaginal secretions can capture exfoliated endometrial cells, allowing for pathological and molecular diagnostics. Though this approach is non-invasive and relatively simple to perform, its sensitivity remains relatively low, which may result in false-negative outcomes, thus limiting its accuracy in detecting early-stage EC. Commonly used method to collect vaginal fluid is tampon, which significantly reduces the pain caused by endometrial sampling. A clinical trial used a visceral analog scale to compare pain associated with tampon collection, tampon brushing, and endometrial biopsy. Concludingly, pain linked to tampon collection was easy to tolerate by the patients while still achieving good diagnostic efficiency^97^-^99^. Moreover, Bakkum-Gamez *et al.* reported that methylation of vaginal fluid collected from tampons for detecting EC exhibited high sensitivity and strong specificity, and adding EDTA buffer to the PBS-based tampon buffer improved sensitivity and specificity^100^.

### 3.2 Endometrial cell collection with special collectors

Endometrial cell collection is the method for direct collection of endometrial cells. The widely used devices to collect endometrial samples include ceramic and pasteurized brushes. The ceramic brush method is one of the most widely used endometrial sampling devices^101^. In a meta-analysis of more than 700 individuals, the ceramic brush method of endometrial cytology sampling was demonstrated to be less invasive, less expensive, and more suitable than endometrial biopsy for screening and diagnosing precancer and malignancy^102^, ^103^. Another study illustrated that the sensitivity of endometrial cells obtained by ceramic brushing was comparable to that of biopsy tissue for detecting atypical hyperplasia and EC^104^ (Table 2). Compared to collecting vaginal fluid, the use of an endometrial brush allows for the direct collection of abnormal cells, offering higher sensitivity and specificity. This approach aids in detecting early cancerous changes and reduces the rate of false-negative results.

### 3.3 The urine collection

Urine testing enables comprehensive screening, improves patient autonomy, and ensures high diagnostic efficiency^105^, ^106^. Urine testing can obtain DNA from locally shed cellular tumors and tumor-free cells, such as cells excreted through the kidneys Second-generation sequencing, microRNA detection, and quantitative SWATH analysis have recently been used to collect patients' urine to diagnose EC^107^-^109^. Wever *et al.* set up a value of urine, cervicovaginal self-sampling, and the clinician's cervical sampling of the three groups of sampling methods to carry out methylation detection^110^. The quantitative methylation-specific PCR was used to detect nine DNA methylation markers; significantly higher methylation levels were found in all groups compared with healthy controls^110^. Another study divided the urine samples into three fractions (whole urine, urine sediment, and urine supernatant) and analyzed DNA methylation markers. All DNA methylation markers exhibited increased methylation levels in all urine fractions of EC patients compared to healthy controls^111^. Whole urine samples demonstrated the highest ability to discriminate patients from controls^111^. However, using urine specimens for EC diagnostic studies is not widely used as compared to vaginal fluid specimens, possibly due to the inability to directly obtain endometrial cells, while its screening and diagnostic accuracy remain to be discussed.

### 3.4 Patient blood or plasma samples collection

Patient blood or plasma samples allow monitoring of small fragments of DNA shed into the bloodstream by the tumor cells, including circulating extracellular nucleic acid, circulating tumor DNA (ctDNA), and circulating tumor cells^112^. DNA from these samples is analyzed to detect point mutations, copy number alterations, gene fusions, and DNA methylation. This method demonstrated good applicability for cancer diagnosis, determining prognosis, targeting gene-specific therapies, monitoring/predicting disease recurrence, and treatment response^113^. Researchers have established the digital droplet PCR (ddPCR) method to detect hypermethylated ctDNA in plasma of EC patients with high analytical specificity and sensitivity in retrospective and cohort studies^114^. The ddPCR exhibited good clinical diagnostic performance in other cancer studies, such as ovarian and colorectal cancer^115^. However, diagnosis of EC by detecting DNA methylation in blood has not been studied on a large scale in clinical settings. Early detection of EC by using blood samples may not be suitable for screening of earlier stage of EC.

Despite various methods for EC screening via DNA methylation testing, a single test cannot fully replace cervical biopsy. Thus, a combined approach is needed to enhance screening accuracy. Herzog *et al.* focused on specific DNA regions within genes such as ZSCAN12 and GYPC using qMSP (quantitative methylation-specific PCR) in the WID quantitative EC (WID-qEC) assay, demonstrating high sensitivity and specificity in detecting different stages and types of EC^20^. Therefore, we should not be limited to DNA methylation alone. Diversifying biomarker detection and combining multiple methods can significantly improve screening accuracy.

## 4. Conclusion

Epigenetics and DNA methylation are novel and promising techniques for biomarker discovery and subsequent screening. Genome-wide and site-specific methylation assays play an important role in screening potential cancer gene methylation sites and in the targeted assessment of gene methylation levels. Clinical studies in EC have reported that detecting single or combined gene methylation markers is comparable to the diagnostic accuracy of endometrium biopsy. It is more acceptable to patients due to its convenience, speed, and painless sampling. However, there are many inconsistencies regarding the results of DNA methylation abnormalities of these tumor suppressor genes in EC. This is due to environment, lifestyle, and individual differences. Additionally, aside from the currently popular cancer gene methylation markers, most gene methylation studies are in the controversial stage. Large numbers of clinical samples are needed to validate and resolve controversies. In conclusion, research on DNA methylation could provide rich and complex information on epigenetic gene regulation in EC and its precursors and offer technical support for better and faster methylation assays in EC screening and diagnosis.

## Funding

Project supported by Capital's Funds for Health Improvement and Research (No. 2024-2-5081).

## Figures and Tables

**Table 1:** Sensitivity, specificity, and AUC values for using gene methylation to diagnose EC

Gene	AUC	Sensitivity	Specificity	Reference
Single-gene				
CDO1	0.842-0.968	82.0%	93.8%	66, 50, 116
BHLHE22	0.95	83.7%	93.7%	50
CELF4	0.94	96.0%	78.7%	50
ZNF662	0.89	92.0%	80%	50
ZNF454	0.938	79.55%	93.42%	50
CDH13	0.67-0.88	81.36%	/	37, 117, 118
RASSF1A	0.75	85.4%	70%	37, 117, 119
CELF4	0.96	96.0%	78.7%	31
HAND2	0.91	/	/	28
ROR2	0.665	/	/	66
EDNRB	0.845	/	/	66
NDN	0.985	/	/	66
DCAF12L1	0.704	/	/	120
MSX1	0.73	/	/	120
Multi-gene				
CDO1+CELF4	/	87.5%	90.8%	50
CDO1+BHLHE22	0.86	92.9%	77.7%	121
BHLHE22+CDO1+HAND2	/	87%	86%	46
BHLHE22+CDO1+TBX5	/	89.1%	88%	46
CDO1+CELF4+ BHLHE22	/	91.8%	95.5%	50
CDO1+ ZNF454	0.931	90.91%	86.84%	116
EMX2OS+NBPF8+ SFMBT2	0.98	97%	97%	99
DCAF12L1+MSX1	0.867	/	/	120

**Table 2:** Changes in AUC values across genes for different clinical modes of endometrial sampling.

Gene	Vaginal fluid (Tao brush)	Vaginal fluid (Pap brush)	Vaginal fluid (tampons)	Urine	Blood
BHLHE22	0.878				
CDO1	0.842				
TBX5	0.703				
HAND2	0.767				
MME	0.7				
PCDHGB 7	0.83	0.86			
HIST1H4F		0.951			
RASSF1		0.938	0.75		
HTR1B			0.82		
HOXA9			0.74		
GHSR				0.95	
SST				0.92	
ZIC1				0.86	
ZSCAN12/OXT					0.99
MLH1					0.96
CDH13			0.67		
ADCYAP1	0.86		0.67		
ASCL2	0.86		0.68		
HS3HT2	0.81		0.67		
HTR1B	0.8		0.82		
MME	0.68		0.7		
NYP	0.86		0.66		
GTF2A1	0.76		0.43		
HAAO			0.68		
HSP2A			0.54		
Reference	46, 122-124	122, 123	37	111	114, 125
